# Synthesis of Pt@TiO_2_@CNTs Hierarchical Structure Catalyst by Atomic Layer Deposition and Their Photocatalytic and Photoelectrochemical Activity

**DOI:** 10.3390/nano7050097

**Published:** 2017-04-29

**Authors:** Shih-Yun Liao, Ya-Chu Yang, Sheng-Hsin Huang, Jon-Yiew Gan

**Affiliations:** Department of Materials Science and Engineering, National Tsing Hua University, Hsinchu 300, Taiwan; syliao@livemail.tw (S.-Y.L.); yachu.y@gmail.com (Y.-C.Y.); huang.sheng.sin@gmail.com (S.-H.H.)

**Keywords:** CNT, ALD, co-catalyst, TiO_2_, photodegradation

## Abstract

Pt@TiO_2_@CNTs hierarchical structures were prepared by first functionalizing carbon nanotubes (CNTs) with nitric acid at 140 °C. Coating of TiO_2_ particles on the CNTs at 300 °C was then conducted by atomic layer deposition (ALD). After the TiO_2_@CNTs structure was fabricated, Pt particles were deposited on the TiO_2_ surface as co-catalyst by plasma-enhanced ALD. The saturated deposition rates of TiO_2_ on a-CNTs were 1.5 Å/cycle and 0.4 Å/cycle for substrate-enhanced process and linear process, respectively. The saturated deposition rate of Pt on TiO_2_ was 0.39 Å/cycle. The photocatalytic activities of Pt@TiO_2_@CNTs hierarchical structures were higher than those without Pt co-catalyst. The particle size of Pt on TiO_2_@CNTs was a key factor to determine the efficiency of methylene blue (MB) degradation. The Pt@TiO_2_@CNTs of 2.41 ± 0.27 nm exhibited the best efficiency of MB degradation.

## 1. Introduction

TiO_2_ has been extensively studied in contaminants degradation and hydrogen generation because of its chemical stability, non-toxicity, and low cost since TiO_2_ electrode was discovered that photodecomposition of water could be achieved by illumination of UV light in 1972 [1], However, TiO_2_ has not been widely applied to the environmental industry because the photoexcited electrons and holes inefficiently diffuse to the surface for redox reaction due to high recombination rate of electrons and holes [2,3,4]. In addition, bulk TiO_2_ suffers from the property of low surface area and then limits its photocatalytic reactivity [5,6]. Although the nanosize of TiO_2_ was fabricated by wet chemical process to increase the surface area of TiO_2_ [4], the TiO_2_ particles aggregated during phase transformation [7]. In order to improve the efficiencies of semiconductor photocatalyst, CNTs as a template to coat with metal oxides (MOs) have attracted great attention in environmental and energy applications because of several reasons. First, CNTs facilitate high specific surface area for MOs deposition and prevent MOs from agglomeration during annealing due to its 1-D structure. Second, CNTs as sensitizer can enhance the photodegradation reaction. Third, heterojunction of MOs@CNTs provides the interface to lower the recombination rate of photo-induced electron-hole pair [8,9,10]. Therefore, TiO_2_-based structure such as TiO_2_/CNTs is a good complementary approach to achieve higher quantum efficiency than that of pure TiO_2_ [11].

Although the TiO_2_/CNTs heterojunction is considered as a good candidate for increasing the quantum efficiencies [11], the effect of MO@CNTs hierarchical catalysts for photodegradation is still limited. For improving the performance of hierarchical catalysts, additional loading with noble metal such as Pt is a good approach because Pt provides more reactive sites in photoreaction as a co-catalyst [12]. However, uniform and well-dispersed Pt particles with precisely controllable size on TiO_2_ are hard to achieve because the deposition usually involves chemical wet processes, such as photo-deposition [13] and impregnation [14], which have some limitations. For instance, for Pt@TiO_2_@carbon composites, the loading of Pt is random through photo-deposition because it is difficult to obtain uniformly reacted surface of TiO_2_ with Pt precursors due to the limitation of irradiation [15]. The agglomeration of Pt particles fabricated by impregnation method takes place when the reduction of metal ions is conducted by chemical reagents or reductive atmosphere such as H_2_ and NH_3_ at high temperatures [16,17]. In comparison to other techniques, atomic layer deposition (ALD) provides excellent thickness control and nearly perfect conformal coverage of complex nanostructures as a result of the surface-controlled growth [18]. Besides, ALD has been confirmed not only to prepare thin films with high aspect ratio but also to fabricate particle catalyst in nanoscale [19]. For example, Pt nanoparticles as a catalyst are well distributed on CNTs with controllable loading and size for proton exchange membrane fuel cell (PEMFC) [20,21]. The effectively decreasing the loading of Pt by ALD to meet the commercial requirement of PEMFC was achieved by the evidence of highly specific power density.

In this study, pristine CNTs were firstly functionalized by temperature-assisted acid-treatment, and then TiO_2_@CNTs structures were prepared by ALD. Pt nanoparticles were subsequently deposited on the TiO_2_@CNTs structures by plasma-enhanced ALD (PEALD) to form Pt@TiO_2_@CNTs composites. Characterization and photocatalytic degradation of methylene blue (MB) by the nanostructures were also investigated.

## 2. Experimental

### 2.1. Acid Treatment of CNTs

Multiwalled CNTs (3–12 μm in length, 20–40 nm in outer diameter, 90% purity) were acquired from Powertip Technology Corporation. The pristine CNTs (p-CNTs) were purified and functionalized by soaked in HNO_3_ (65% purity) and then refluxed at 140 °C for 6 h to obtain acid-treated CNTs (a-CNTs). The a-CNTs were washed with deionized water and alcohol until pH value was 7, collected by centrifuge, and then dried at 80 °C for 24 h. The detail of sample preparation has been reported in the previous work [22].

### 2.2. Fabrication of TiO_2_@CNTs Structure by ALD

The sample of a-CNTs was used as a support for coating with TiO_2_ by ALD. Firstly, 5 mg of CNTs were sonicated in 15 mL N-Methyl-2-pyrrolidone for 60 min to get good dispersion on a Si wafer. The dispersed CNTs were then deposited with TiO_2_ at 300 °C by ALD using TiCl_4_ and H_2_O as the precursors. The temperature of precursors was maintained in room temperature. Each cycle consisted of a precursor pulse for 0.08 s and a purge with N_2_ for 7 s. The number of precursor/purge cycles was from 25 to 200 cycles of ALD to achieve precise control of the particles size of TiO_2_.

### 2.3. Fabrication of Pt@TiO_2_@CNTs Composites by PEALD

Pt particles were then deposited on the as-prepared TiO_2_@CNTs composites by PEALD using (methylcyclopentadienyl) trimethylplatinum (MeCpPtMe_3_) and oxygen plasma as precursors [23]. The sequence of Pt-N_2_-O_2_ plasma-N_2_ was 0.5-5-3-5 s, respectively. The power of induced couple plasma was 400 W and substrate temperature was 300 °C. The temperature of Pt precursor is heated to 80 °C for enough vapor pressure. The cycles of PEALD were from 10 to 100 to meet precisely controllable size and loading of Pt particles.

### 2.4. Photocatalytic Activity and Characterization

The surface chemistry was analyzed by Fourier-Transform Infrared Spectrometer (FTIR, Bruker, Vertex 80v and Tensor 27, Billerica, Massachusetts, USA). The TiO_2_@CNTs and Pt@TiO_2_@CNTs composites prepared by using a-CNTs as the template were examined by transmission electron microscopy (TEM, JEOL 2100F, Akishima, Tokyo, Japan). The structural analysis was performed by X-ray diffraction (XRD, Shimadzu 6000, Nakagyo-ku, Kyoto, Japan) with the Cu K_α_ radiation. The optical properties were obtained by photoluminescence spectroscopy (PL, Horiba Jobin Yvon, Labram HR 800, Minami-Ku, Kyoto, Japan) with excitation wavelength of 325 nm (He-Cd laser, Kimon IK3301R-G Itabashi-Ku, Kyoto, Japan), laser power of 30 mW, and a spot size of 0.79 μm^2^. The photocatalytic activities of all the samples were examined by studying the MB degradation with 200 W Hg lamp as irradiation source. The initial concentration of MB is 6.26 × 10^−5^ M. The distance between photoreactor and lamp was 10 cm. 2 mg of prepared samples were added to the MB solution and P25 was tested as control group. The absorption of MB solution at 664 nm was measured by a UV–Visible spectrometer (Hitachi U-3010, Chiyoda-ku, Tokyo, Japan) for every 10 min illumination until 60 min. The total organic carbon (TOC) concentration was determined with a TOC analyzer (Shimadzu TOC-5000, Nakagyo-ku, Kyoto, Japan). Photoelectrochemical cell (PEC) measurement with 1 M KOH as electrolyte was performed in a three electrode electrochemical cell. Ag/AgCl electrode and Pt foil were used as the reference electrode and counter electrode, respectively. All the samples prepared for PEC measurement were dispersed in polyethylene glycol to form slurry and subsequently printed onto fluorine-doped tin oxide (FTO) by doctor-blade technique as working electrodes [24]. The procedure was repeated to obtain the film thickness of 3 μm that was determined by alpha-step profilometer (Veeco Tektak 150, Plainview, New York, NY, USA). The photocurrent was measured by a potentiostat (Solartron 1286, Bognor Regis, West Sussex, UK) by illumination with a 200 W Hg lamp at a sweeping rate of 10 mV·s^−1^ from −1.0 to 0.4 V. The active area was 1 cm × 1 cm.

## 3. Results and Discussion

### 3.1. Modification of Multiwall CNTs

**Figure 1:**
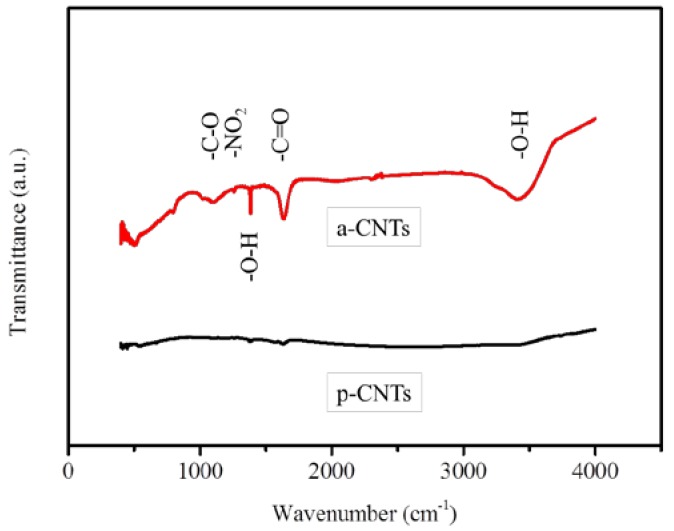
FTIR spectra of pristine and acid-treated CNTs. The abbreviations of a-CNTs and p-CNTs correspond to acid treatment of CNTs for 6 h by nitric acid and pristine CNTs, respectively.

**Figure 2:**
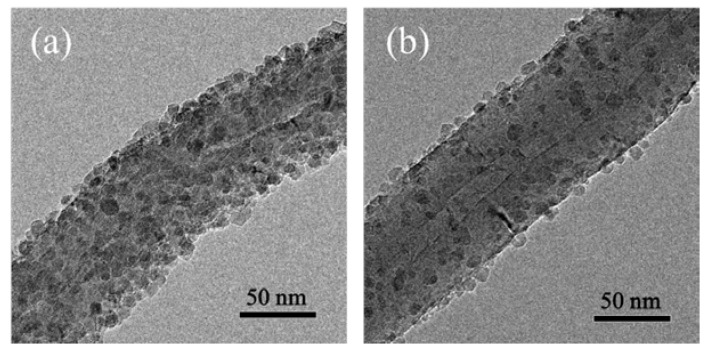
TEM morphologies of TiO_2_ deposited on (**a**) a-CNTs and (**b**) p-CNTs by 50 cycles of ALD at 300 °C.

Figure 1 shows the FTIR spectra of p-CNTs and a-CNTs. The FTIR peaks are identified for different types of organic functional groups. The small peak at 1040 cm^−1^ can be related to the C–O stretching vibration of either alcohol or carboxyl groups [25]. The peak at 1280 cm^−1^ is attributed to the nitrates NO_2_ symmetric stretching bands [26]. The peak at around 1400 cm^−1^ is possibly associated with O–H bending deformation of carboxyl groups and the C=C stretching bands placed by the functional groups. The absorption peaks at around 1700 cm^−1^ is ascribed to the C=O stretching vibration of carboxyl groups [26]. A clear broad peak at around 3400 cm^−1^ can be referred to O–H stretching. The outcome of FTIR analysis suggests that oxidation has conducted by the acid treatment and demonstrates that there is significant difference between p-CNTs and a-CNTs. Figure 2 shows the morphology of TiO_2_ deposited both p-CNTs and a-CNTs by 50 ALD cycles at 300 °C. There was more TiO_2_ particles deposited on the surface of a-CNTs than that on p-CNTs. The coverage of TiO_2_ on a-CNTs was more complete because of higher oxygen-containing functional on the surface of a-CNTs. This suggests that the functional groups played an important role in the density of TiO_2_ particles deposited on the CNTs, resulting in that the TiO_2_ particles coated on a-CNTs were more uniform and compact than that on p-CNTs. This implies the nucleation sites of TiO_2_ on a-CNTs were higher than that on p-CNTs after acid treatment. However, the particle size of TiO_2_ deposited on both p-CNTs and a-CNTs was not obviously different to each other. In order to increasing the amount of TiO_2_ particles loaded on the CNTs for higher efficiency of MB photodegradation, the acid-treated CNTs for 6 h was choose to be the support as following samples prepared by ALD to form TiO_2_@CNTs composites.

### 3.2. Fabrication and Characterization of TiO_2_@CNTs Structures

**Figure 3:**
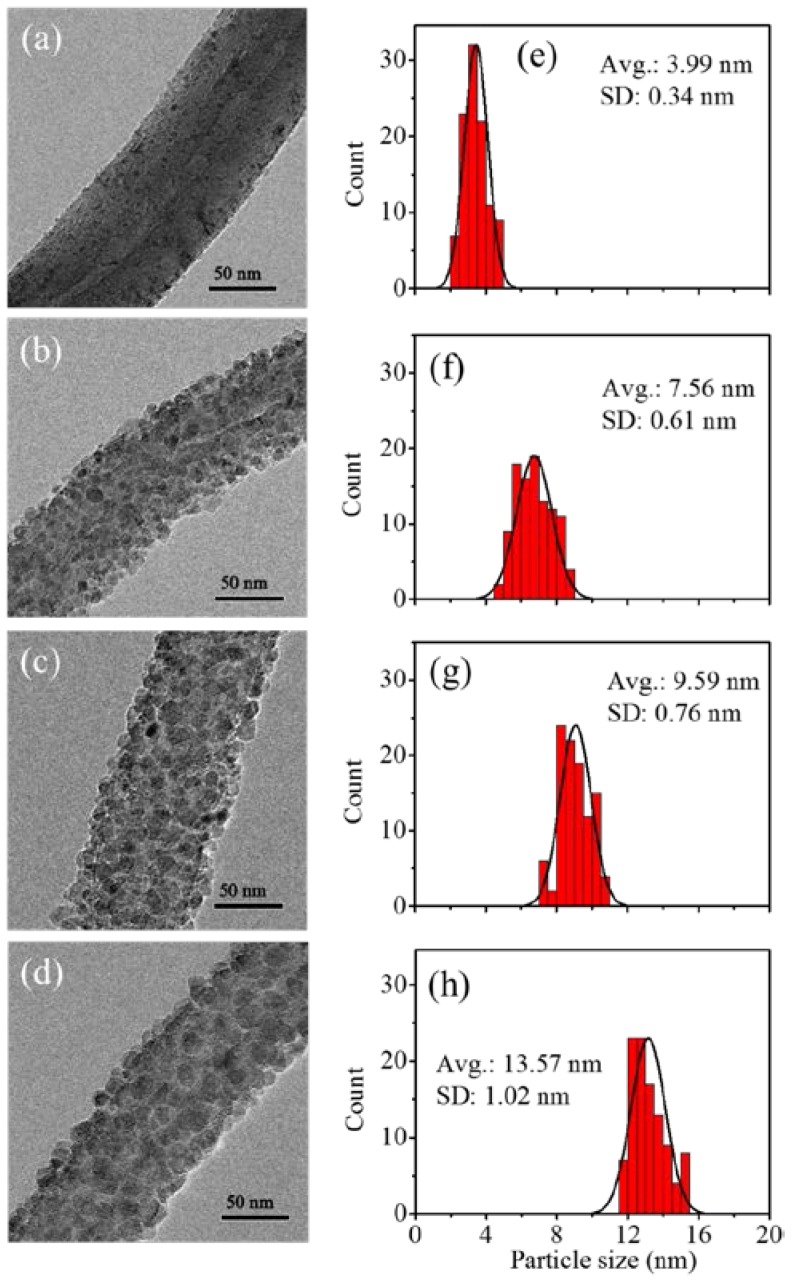
TEM images of TiO_2_ deposited on a-CNTs with (**a**) 25; (**b**) 50; (**c**) 100; and (**d**) 200 cycles of ALD at 300 °C. The corresponding figures (**e**–**h**) on the right hand side are the distribution of TiO_2_ particles.

**Figure 4:**
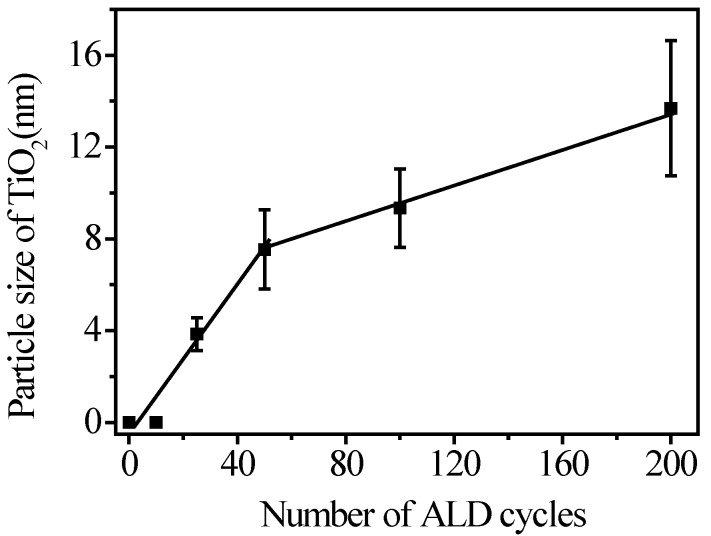
Variation of TiO_2_ particle size on a-CNTs with ALD cycle number.

The difference of TiO_2_ particle size grown by various ALD cycles at 300 °C was observed by TEM, as shown in Figure 3. It is worthy to note that the particle size distribution of TiO_2_ fabricated by variation of ALD cycles was uniform and close to Gaussian distribution. The deviation of average size of TiO_2_ is less than 8%. This shows that the TiO_2_ particles could be precisely and uniformly coated on the surface of a-CNTs by ALD to synthesize complete TiO_2_@CNTs heterojunction structures. By counting more than one hundred particles from the TEM images, the average diameters of TiO_2_ particles are from 3 to 13 nm and plotted in Figure 4. The particle size increases as the cycle number increases, and the relation can be divided into two regions from Figure 4. Based on the generally accepted mechanism of ALD using TiCl_4_ and H_2_O as precursors [27], the process can be separated to two half reactions for a typical self-terminating process in ALD. In the very beginning, TiCl_4_ reacts with the surface OH groups and then releases HCl in the first-half reaction:

n(-OH)_(s)_ + TiCl_4(g)_ → (-O-)nTiCl_4-n(s)_ + nHCl_(g)_ (1)

In which (s) presents the surface. The nonstoichiometric titanium chloride (TiCl*_x_*) species adsorbed on the surface may react with water and then release HCl in the second-half reaction, which results in the recovery of the surface OH groups again for the next cycle:

(-O-)nTiCl_4-n(s)_ + (4−n)H_2_O_(g)_ → (-O-)nTi(OH)_4_^−^_n(s)_ + (4−n)HCl_(g)_ (2)

For TiO_2_@CNTs composites, once the TiCl*_x_* species has formed on the surface of CNTs, the H_2_O molecules then reacted with TiCl*_x_* to form TiO_2_ and subsequently left the OH functional groups again in the surface. The surface functional groups on a-CNTs would affect the growth rate of TiO_2_ particles because the reaction was determined by the interaction among surfaces of CNTs, TiCl_4_ molecules and H_2_O molecules. There was a substrate-enhanced growth process with a higher growth rate when the ALD cycles were less than 50 [28]. In this region, the surface a-CNTs with fully oxygen-containing functional groups enhanced the TiO_2_ particles to grow. However, that particles were probably too tiny to be observed when the cycle number was less than 25. After 50 cycles of ALD, TiO_2_ was deposited on the existing particles, which resulted in a linearly lower growth rate in the subsequent cycles due to consuming out of the initial functional groups. The growth rates were calculated to be 1.5 Å/cycle and 0.4 Å/cycle for substrate-enhanced growth process and linear growth process, respectively.

**Figure 5:**
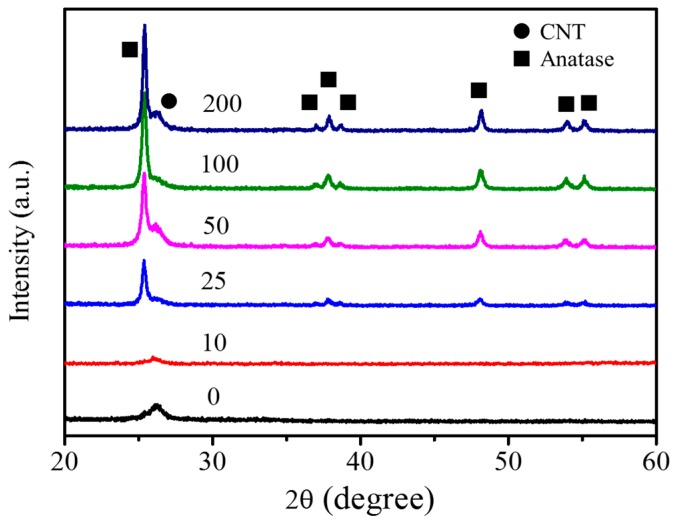
XRD patterns of TiO_2_@CNT composites with various ALD cycles at 300 °C.

The XRD patterns in Figure 5 show the phase of TiO_2_ deposited on a-CNTs by variable ALD cycles. The TiO_2_ particles with different ALD cycles deposited on a-CNTs exhibited a crystalline anatase phase. The intensity of anatase phase increases as the ALD cycles increase from 25 to 200. Rutile phase was not observed in the TiO_2_ particles coated on a-CNT at 300 °C. However, the crystallinity of TiO_2_ was hardly observed when the number of cycle was less than 25. According to Zhang’s report [29], the nucleation of TiO_2_ ALD was about 20 cycles. The size of TiO_2_ nuclei was less than 2 nm when temperatures were 120 °C and 200 °C. In the meanwhile, the crystalline particles were also consisted of amorphous shell and crystalline core. The ratio of amorphous shell and crystalline core decreased as ALD cycles decreased [29]. The TiO_2_ particles less than 25 ALD cycles were probably too weak to be observed in XRD patterns due to the initial nucleation of TiO_2_. It was in agreement with the TEM analysis, as shown in Figure 3a. Although TiO_2_ deposited on a-CNTs at 300 °C was uniform from 50 to 200 cycles, the TiO_2_ nanoparticles around 10 nm with higher photocatalytic property have been demonstrated [30]. Based on that, the TiO_2_ particles fabricated by 100 ALD cycles with 9.56 nm were chosen for the following characterization and MB photodegradation.

### 3.3. Fabrication and Characterization of Pt@TiO_2_@CNTs Structures

**Figure 6:**
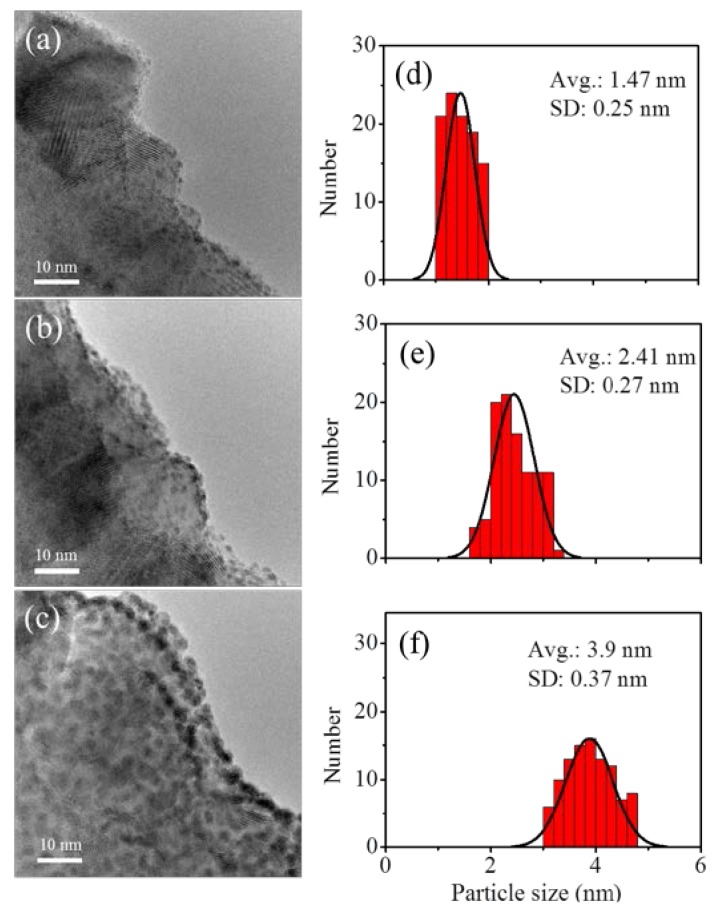
TEM micrographs of Pt particles prepared with (**a**) 25 cycles; (**b**) 50 cycles; and (**c**) 100 cycles of PEALD. The corresponding figures of (**d**–**f**) on the right hand side are the distribution of Pt particles.

**Figure 7:**
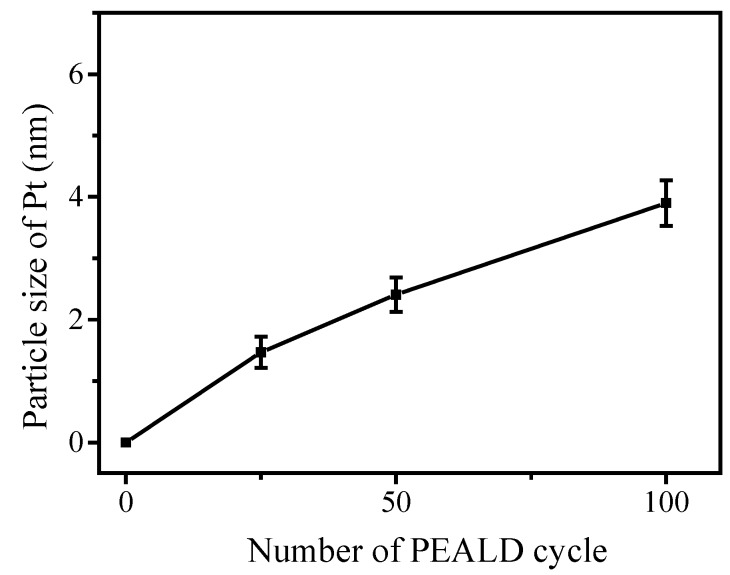
Variation of Pt particle size on TiO_2_@CNTs with PEALD cycle number.

After the TiO_2_@CNTs structure was fabricated, Pt particles as co-catalyst were then deposited on the TiO_2_ surface at 300 °C with 25, 50, and 100 cycles by PEALD using MeCpPtMe_3_ and oxygen plasma as precursors. When the lengths of pulse time for the sequence MeCpPtMe_3_-N_2_-O_2_ plasma-N_2_ were 0.5-5-3-5 s, the saturated deposition rate of Pt on TiO_2_@CNTs was 0.39 Å/cycle. The size distributions and Pt particles by calculating more than one hundred particles from the TEM images are showed in Figure 6. The sizes of Pt particle with 25, 50, and 100 PEALD cycles are 1.47 ± 0.25 nm, 2.41 ± 0.27 nm, and 3.90 ± 0.37 nm, respectively. The particle size of Pt related to number of ALD cycle was shown in Figure 7. From Figure 7, the particle size increases with the cycle number, and the relation only has one slope. In comparison to TiO_2_ fabricated by ALD using TiCl_4_ and H_2_O as precursors, there is no substrate-enhanced growth process with a higher growth rate before having consumed the initial functional groups [28]. Therefore, the growth rate of Pt particles on the TiO_2_ surface is the same as that on Pt particles deposited earlier, which results the slope derived from particle sizes of Pt divided to cycle number of ALD is linear. This implies the growth behavior of Pt in PEALD between the interface of TiO_2_ and Pt precursors is more inert than that of TiO_2_ in ALD between the interface of a-CNTs and TiO_2_ precursors. Generally speaking, the precursors of ALD reacting to surface of substrate can be divided to two steps: one is physisorption and the other is chemsorption. The adsorption can be considered reversible in physisorption, as illustrated in reactions (3) and (4),

(3) $$∥*\;+\;M(g)\overset{r_{a}}{\to }∥*M(g)$$

(4) $$∥*M(g)\overset{r_{d}}{\to }∥*\;+\;M(g)$$

where ∥* is the active surface of substrate for adsorption of precursor molecules. Adsorption rate, *r_a_*, refers to the amount of molecules *M* attached to the surface per unit time. Desorption rate, *r_d_,* denotes to the amount of molecules *M* detached from the surface per unit time. For physisorption, the partial pressure of the reactant is a key parameter to describe the adsorption behavior with three assumptions [28], the monolayer is achieved by the maximum amount of adsorbed molecules, all adsorption sites on the surface are assumed equal, and adjacent molecules adsorbed on the surface are assumed not to interact with each other. Once the physisorption is achieved, the precursor would react with functional groups, dangling bond, and defect on the surface of substrate as chemical bonding so called chemsorption. Therefore, the coverage of adsorbed species denoted as *Q* describing the adsorption process, as following:

(5) $$\frac{dQ}{dt}{=r}_{a}-r_{d\;}{=\;k}_{a}p\left(1-Q\right)\;-{\;k}_{d}Q$$

After saturation in the ALD process, the coverage is constant (d*Q*/d*t* = 0), chemsorption coverage *Q*^eq^ is integrated by the condition of Langmuir isotherm to show the relationship between equilibrium constant of adsorption, *K*, as following:

(6) $$Q^{\mathrm{eq}}=\frac{k_{a}p}{k_{a}{p+k}_{d}}=\frac{1}{1+{\left(Kp\right)}^{-1}}$$

Considering time effect into Equation (5), the chemisorption coverage *Q* is calculated as a function of time,

(7) $${Q=Q}^{\mathrm{eq}}(1\;-{\;e}^{-\left(k_{a}p-k_{d}\right)\;t})$$

However, the three assumptions to derive the Equation (5) are not available in real process. For Pt in PEALD, the molecule of MeCpPtMe_3_ is larger than TiCl_4_. Steric hindrance of the MeCpPtMe_3_ molecules is taken into account. The initially accessible ligands of the chemisorbed species on the surface block up the space for the neighbor reactants [31]. Therefore, the initial *k_a_* of MeCpPtMe_3_ in PEALD is lower than that of TiCl_4_ in ALD. That presumably explains the initial growth rate of Pt is the same as the following PEALD cycles. By contrast, the molecule of TiCl_4_ is smaller and easier to react to surface of a-CNTs without steric hindrance, which causes the initially higher growth rate due to higher adsorption rate. However, once the consumption of functional group on the surface of a-CNTs has been achieved, the TiCl_4_ molecule would deposit on the residual TiO_2_ particle with lower growth rate because molecules of TiCl_4_ reacted to the existed TiO_2_ particles rather than surface of a-CNTs.

### 3.4. Photocatalytic Efficiency of Pt@TiO_2_@CNTs Structure

**Figure 8:**
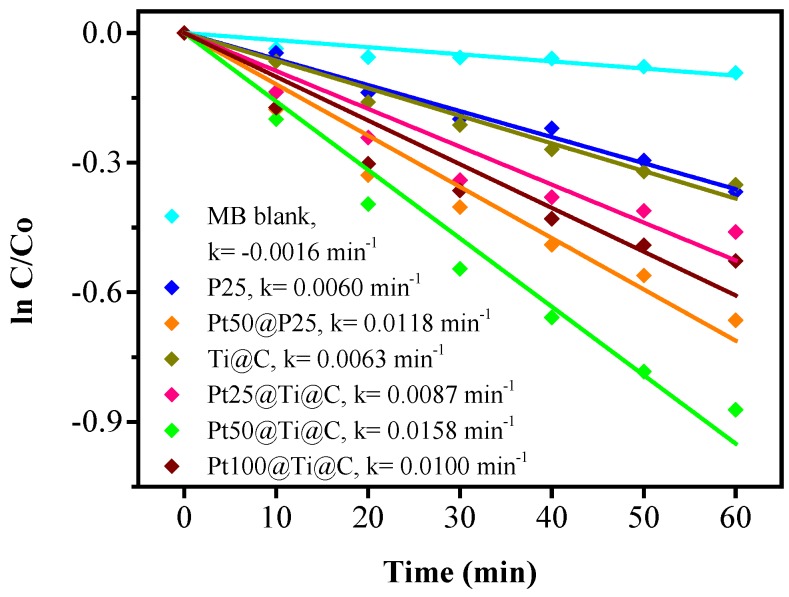
Photodecomposition of MB for P25, Pt50@P25, TiO_2_@CNTs (Ti@C), and Pt@TiO_2_@CNTs with different particle size of Pt (Pt25@Ti@C, Pt50@Ti@C, and Pt100@Ti@C).

The adsorption-desorption equilibrium of MB by the samples has been reached before irradiation. The adsorption capacities of dye molecules for all TiO_2_@CNTs, Pt@TiO_2_@CNTs samples are almost the same. This implies that the adsorption capacity was mainly affected by the CNTs as substrate. Figure 8 displays the photocatalytic activities of the TiO_2_@CNTs of 9.56 nm with 100 ALD cycles at 300 °C (Ti@C) and Pt@TiO_2_@CNTs with the particle size of 1.47 ± 0.25 nm, 2.41 ± 0.27 nm, and 3.90 ± 0.37 nm fabricated by 25, 50, and 100 PEALD cycles at 300 °C, respectively (Pt25@Ti@C, Pt50@Ti@C, and Pt100@Ti@C). P25 and Pt-loaded P25 with 50 PEALD cycles (Pt50@P25) were also tested for control specimens. The efficiency of MB degradation can be fitted by a pseudo first-order kinetics with different particle size of Pt [32], as follows:

(8) $${C\;=\;C}_{o}\;\exp\;(-kt)$$

where *C* is the concentration of MB after reaction time *t*, C_o_ is the initial concentration of MB, and *k* is the rate constant. According to the photodecomposition result, the *k* values for each TiO_2_@CNTs and Pt@TiO_2_@CNTs samples were calculated from the slopes, as shown in Figure 8. A blank test reveals that the MB is slowly degraded by the irradiation after 60 min. The kinetic rate constant *k* for the blank test is 0.0016 min^−1^. The TiO_2_@CNTs composite and P25 have almost the same efficiency for MB degradation with the *k* values being 0.0063 and 0.0060 min^−1^, respectively. The *k* value of Pt@TiO_2_@CNTs with Pt particles of 1.47 ± 0.25 nm by 25 cycles is 0.0087 min^−1^. As the particle size of Pt increases to 2.41 ± 0.27 nm by 50 cycles, the photodegradation efficiency substantially increases with rate constant raising to 0.0158 min^−1^. That would presumably is due to a large amount of Pt for MB decomposition. However, as the particle size of Pt increases to 3.90 ± 0.37 nm by 100 cycles, the rate constant reduces to 0.010 min^−1^. To sum up, the samples of Pt@TiO_2_@CNTs with different Pt loading have a higher decomposition rate of MB than those without Pt loading as co-catalyst. Pt particles provide more effective reaction sites for reductive reaction and cause formation of Schottky barrier between the interface of Pt and TiO_2_. Once the interfacial charge transfer was facilitated, the recombination rate of electrons and holes would become lower and then result in improving separation of electrons and holes [33]. The Pt@TiO_2_@CNTs with Pt deposited by 50 PEALD cycles shows the higher *k* value than that of P25 with the same PEALD cycles of Pt. The synergistic effect of Pt loaded on TiO_2_@CNTs is more obvious than that of P25.

**Figure 9:**
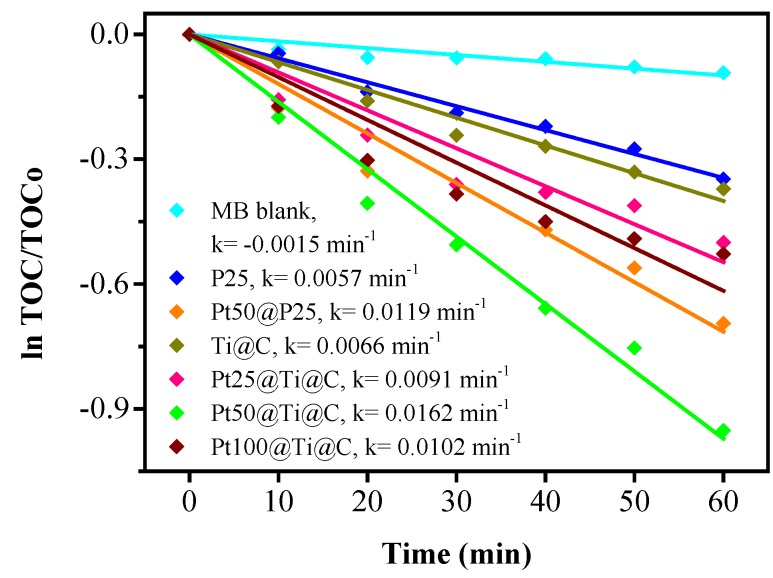
Disappearance of the TOC during the photocatalytic degradation of MB for P25, Pt50@P25, TiO_2_@CNTs (Ti@C), and Pt@TiO_2_@CNTs with different particle size of Pt (Pt25@Ti@C, Pt50@Ti@C, and Pt100@Ti@C).

Figure 9 shows the change of TOC for the photocatalytic degradation of MB during UV irradiation with all samples as photocatalysts, which is consistent with the tendency shown in Figure 8. The gradual decrease of TOC represented the gradual disappearance of organic carbon when the MB solution which contained photocatalysts was exposed under UV irradiation. The *k* values calculated from TOC were 0.0060, 0.0118, 0.0063, 0.0087, 0.0158, or 0.0100 min^−1^ with P25, Pt50@P25, Pt25@Ti@C, Pt50@Ti@C, or Pt100@Ti@C after UV irradiation for 60 min.

**Figure 10:**
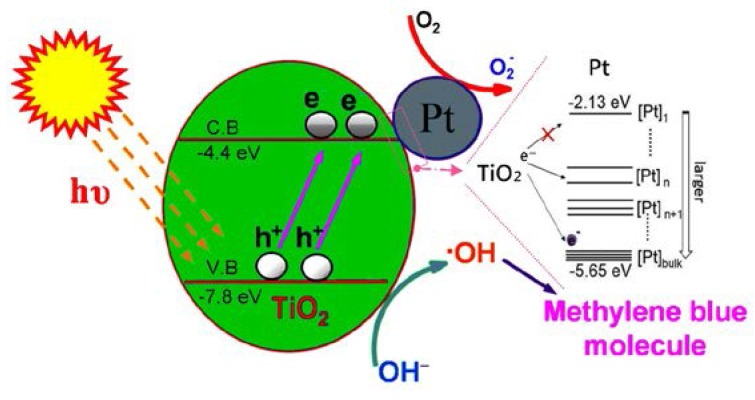
Schematic diagram of the energy levels of Pt particles with different sizes and the energy band of TiO_2_ after irradiation.

Interestingly, the reason why the efficiency of Pt@TiO_2_@CNTs with Pt particles of 2.41 ± 0.27 nm is higher than that of 3.90 ± 0.37 nm deserves further investigation. As a whole, effective electron-hole separation is related to the loading, size, and distribution of Pt particles on TiO_2_ [34,35]. From the view of photocatalytic reaction, Pt particles of several nanometers can enhance activity of photodegradation. However, larger Pt particles may provide more recombination sites for photo-generated electrons and holes [35,36]. In addition, not only surface reactive site on TiO_2_ but also the light absorption of TiO_2_ are reduced by the too high Pt loading [34]. Therefore, optimum size, loading, and uniform distribution of Pt particles on TiO_2_ are necessary to obtain effective electron-hole separation and to achieve a higher photocatalytic activity. Pt particles with 0.2 nm different size could particularly increase the efficiency of photoreduction of CO_2_ to CH_4_ [37]. The conversion rate increases almost twice within only 0.2 nm different of Pt particles. This implies that a 1–2 nm different particle size in this study may make a big difference in the MB degradation. Based on quantum confinement effect, decreased particle size of Pt has larger energy band gap [37]. Pt particles have higher energy band separation and prevent electron transfer from TiO_2_ conduction band to Pt when the particles are extremely small. However, electrons can easily transfer to Pt particles with a suitable energy level lower than −4.4 eV when the particle size of Pt increases [37], as shown in Figure 10. If the size becomes too larger, the behavior of Pt particles is more like bulk Pt due to energy level narrowing, which increases the recombination rate of electrons and holes [36]. To sum up, the Pt particles with 2.41 ± 0.27 nm reconcile the quantum confinement effect with suitable energy band gap and avoid the behavior of bulk Pt as recombination center of electrons and holes. This presumably explains that the sample with Pt particles of 2.41 ± 0.27 nm shows the highest efficiency for MB degradation. Table 1 summarizes the particle sizes of Pt for the highest reaction rate of some applications from the literature. The optimum particle size of Pt in this study is 2.41 ± 0.27 nm for degradation of MB, compared to 1.9, 2, 1.75, 2, and 2.2 nm for applications of C_2_H_4_ oxidation, glycerol reforming, water splitting, and CO oxidation by using different process for deposition of Pt, respectively. The all show an optimum particle size close to 2 nm. Therefore, for the Pt@TiO_2_@CNTs structure with the size of 2.41 ± 0.27 nm of Pt for superior efficiency of MB degradation is probably because of its high area and appropriate diffusion length for electrons and holes.

**Figure 11:**
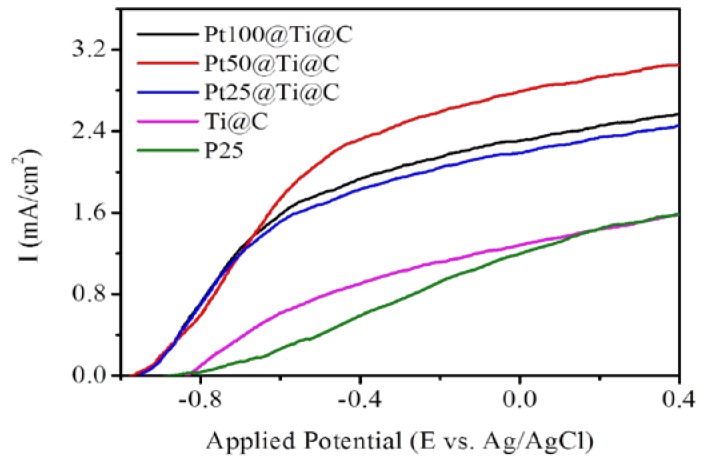
Current-voltage characteristics of P25, TiO_2_@CNTs (Ti@C), and Pt@TiO_2_@CNTs with different particle size of Pt (Pt25@Ti@C, Pt50@Ti@C, and Pt100@Ti@C).

Besides, it is well know the activity of photocatalyst can be demonstrated by the PEC analysis, as shown in Figure 11. In comparison to pure P25 and TiO_2_@CNTs, the open circuit potential (*E*_ocp_) of the Pt@TiO_2_@CNTs with different particle sizes of Pt are all −951 mV (versus Ag/AgCl) with negatively shifted 130 mV. The lower open circuit potential implies that Pt@TiO_2_@CNTs has less recombination sites than that of TiO_2_@CNTs and P25, which results in higher photocurrent density and higher photodecomposition rate of MB [38]. The photocurrent of the Pt@TiO_2_@CNTs with Pt particles of 2.41 ± 0.27 nm has higher photocurrent densities than those other samples. The result is consistent to the MB degradation and shows that the optimized particle size of Pt plays an important role for design of noble metal as co-catalyst to improve efficiency of photocatalyst.

**Figure 12:**
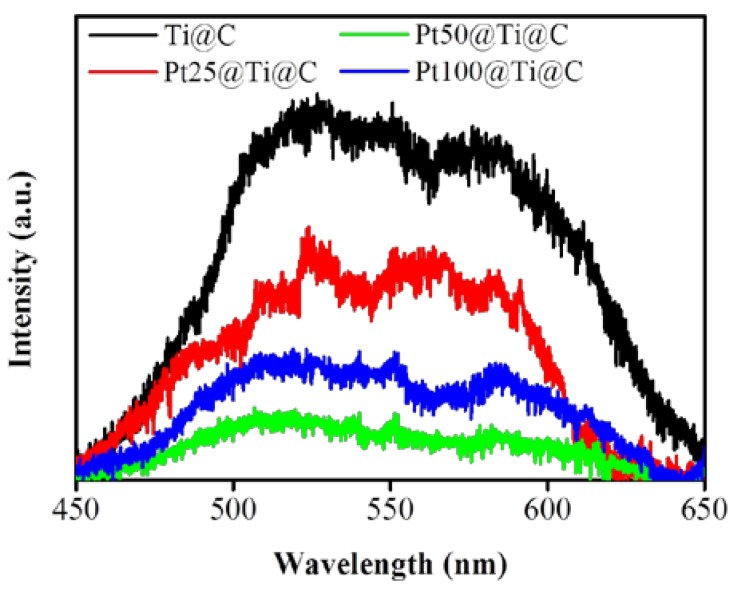
PL spectra of TiO_2_@CNTs (Ti@C) and Pt@TiO_2_@CNTs with different particle size of Pt (Pt25@Ti@C, Pt50@Ti@C, and Pt100@Ti@C).

Furthermore, the PL spectra are useful to study the efficiency of trapping, immigration, and transfer of charge carriers for understanding the recombination of electrons and holes because recombination of free charge carries result in signal of PL emissions [39]. Figure 12 shows the PL spectra of TiO_2_@CNTs and Pt@TiO_2_@CNTs with various particle size of Pt are all located at around 505 nm. For TiO_2_, the emission wavelength at around 505 nm is the charge transfer from Ti^3+^ to the oxygen anion in the TiO_6_^8−^ complex [39], and the recombination rate of electrons and holes is related to the intensity of the PL emission [40,41]. Therefore, the samples with the higher intensity of PL emission could be suggested to the existence of more trapping centers that enhance the recombination rate of electrons and holes. For Pt@TiO_2_@CNTs with Pt particles of 2.41 ± 0.27 nm, the lowest intensity proves that the proper particle size of Pt can suppresses the recombination of electrons and holes in comparison to other samples. The result is well in line with the PEC analysis and MB degradation.

## 4. Conclusions

The formation of TiO_2_@CNTs and Pt@TiO_2_@CNTs structures with various ALD and PEALD cycles was studied in detailed. It demonstrated that the CNTs with nitric acid treatment could generated oxygen-containing functional groups on the surface. The density of TiO_2_ particles deposited by ALD is related to the amount of oxygen-containing functional groups on the surface of CNTs. The saturated growth rates of TiO_2_ on a-CNTs were 1.5 Å/cycle and 0.4 Å/cycle, which result from substrate-enhanced process and linear process, respectively. Compared to TiO_2_, the saturated growth rate of Pt on TiO_2_@CNTs was linearly 0.39 Å/cycle due to the steric hindrance of Pt precursors. With increasing the ALD cycles, the as-deposited TiO_2_ particles all show anatase phase and increase the intensity of crystallinity at 300 °C. The composite with Pt particles of 2.41 ± 0.27 nm has the highest efficiency MB degradation because it reconciles the quantum confinement effect with suitable energy band gap and avoid the behavior of bulk Pt as recombination center of electrons and holes. In comparison to TiO_2_@CNTs, the samples with Pt loading show the relatively negative *E*_ocp_ and higher photocurrent due to the Pt particles as co-catalyst. The result of PL spectra proved the lower electrons and holes recombination rate with optimum particle size of Pt. For Pt@TiO_2_@CNTs of 2.41 ± 0.27 nm, the higher photocurrent and lowest intensity of PL emission all demonstrate lower recombination rate of electrons and holes in agreement with MB degradation. The key achievements of this study are the precise control of particle sizes of TiO_2_ and Pt and quantification of the photocatalytic efficiencies of TiO_2_@CNTs coupled with Pt particles, which provide useful information for further design of Pt@TiO_2_@CNTs structure for catalytic, sensor, and photochemical applications.

## Figures and Tables

**Table 1:** Summary of particle sizes of Pt for the highest photodegradation rate.

Size Distribution (nm)	Optimized Size (nm)	Preparation Method ^a^	Test Reagent	Platinum Precursor	Ref.
1.9–6.7	1.9	Hydrothermal	CO, C_2_H_4_	PtCl_4_	[42]
1.2–4.2	2	Impregnation	Glycerol	H_2_PtCl_6_·6H_2_O	[43]
1.8–4.9	1.75	MW-solvothermal	CO_2_	H_2_PtCl_6_	[44]
1–15	2	sputter	EtOH/H_2_O	Pt target	[45]
2.2–16.7	2.2	H_2_ reduction	CO_2_	N.A	[46]
1.47–3.9	2.41	PEALD	MB	MeCpPtMe_3_	This study

^a^: MW: microwave assited List of figures.

## References

[1] Fujishima A., Honda K. (1972). Electrochemical photolysis of water at a semiconductor electrode. Nature.

[2] Serpone N., Lawless D., Khairutdinov R., Pelizzetti E. (1995). Subnanosecond relaxation dynamics in TiO_2_ colloidal sols (particle sizes R_P_ = 1.0-13.4 nm). Relevance to heterogeneous photocatalysis. J. Phys. Chem..

[3] Wang C.C., Zhang Z.B., Ying J.Y. (1997). Photocatalytic decomposition of halogenated organics over nanocrystalline titania. Nanostruct. Mater..

[4] Zhang Z.B., Wang C.C., Zakaria R., Ying J.Y. (1998). Role of particle size in nanocrystalline TiO_2_-based photocatalysts. J. Phys. Chem. B.

[5] Parra S., Stanca S.E., Guasaquillo I., Thampi K.R. (2004). Photocatalytic degradation of atrazine using suspended and supported TiO_2_. Appl. Catal. B Environ..

[6] Zhu H.Y., Gao X.P., Lan Y., Song D.Y., Xi Y.X., Zhao J.C. (2004). Photocatalytic degradation of atrazine using suspended and supported TiO_2_. J. Am. Chem. Soc..

[7] Zhang H.Z., Banfield J.F. (2000). Understanding polymorphic phase transformation behavior during growth of nanocrystalline aggregates: Insights from TiO_2_. J. Phys. Chem. B.

[8] Britto P.J., Santhanam K.S.V., Rubio A., Alonso J.A., Ajayan P.M. (1999). Improved charge transfer at carbon nanotube electrodes. Adv. Mater..

[9] Che J.W., Cagin T., Goddard W.A. (2000). Thermal conductivity of carbon nanotubes. Nanotechnology.

[10] Collins P.C., Arnold M.S., Avouris P. (2001). Engineering carbon nanotubes and nanotube circuits using electrical breakdown. Science.

[11] Orlanducci S., Sessa V., Terranova M.L., Battiston G.A., Battiston S., Gerbasi R. (2006). Nanocrystalline TiO_2_ on single walled carbon nanotube arrays: Towards the assembly of organized C/TiO_2_ nanosystems. Carbon.

[12] Li C., Yuan J., Han B., Jiang L., Shangguan W. (2010). TiO_2_ nanotubes incorporated with CdS for photocatalytic hydrogen production from splitting water under visible light irradiation. Int. J. Hydrog. Energy.

[13] Li F.B., Li X.Z. (2002). The enhancement of photodegradation efficiency using Pt-TiO_2_ catalyst. Chemosphere.

[14] Bamwenda G.R., Tsubota S., Nakamura T., Haruta M. (1995). Photoassisted hydrogen-production from a water-ethanol solution: A compasison activities of Au-TiO_2_ and Pt-TiO_2_. J. Photochem. Photobiol. A.

[15] Gomes H.T., Machado B.F., Silva A.M.T., Dražić G., Faria J.L. (2011). Photodeposition of Pt nanoparticles on TiO2–carbon xerogel composites. Mater. Lett..

[16] Chen M.-L., Zhang F.-J., Oh W.-C. (2010). Preparation and Catalytic Properties of Pt/CNT/TiO2 Composite. J. Korean Ceram. Soc..

[17] Bedolla-Valdez Z.I., Verde-Gómez Y., Valenzuela-Muñiz A.M., Gochi-Ponce Y., Oropeza-Guzmán M.T., Berhault G., Alonso-Núñez G. (2015). Sonochemical synthesis and characterization of Pt/CNT, Pt/TiO2, and Pt/CNT/TiO2 electrocatalysts for methanol electro-oxidation. Electrochim. Acta.

[18] Marichy C., Pinna N. (2013). Carbon-nanostructures coated/decorated by atomic layer deposition: Growth and applications. Coordin. Chem. Rev..

[19] Liu C., Wang C.C., Kei C.C., Hsueh Y.C., Perng T.P. (2009). Atomic layer deposition of platinum nanoparticles on carbon nanotubes for application in proton-exchange membrane fuel cells. Small.

[20] Hsueh Y.C., Wang C.C., Kei C.C., Lin Y.H., Liu C., Perng T.P. (2012). Fabrication of catalyst by atomic layer deposition for high specific power density proton exchange membrane fuel cells. J. Catal..

[21] Hsueh Y.C., Wang C.C., Liu C., Kei C.C., Perng T.P. (2012). Deposition of platinum on oxygen plasma treated carbon nanotubes by atomic layer deposition. Nanotechnology.

[22] Huang S.H., Wang C.C., Liao S.Y., Gan J.Y., Perng T.P. (2015). CNT/TiO_2_ core-shell structures prepared by atomic layer deposition and characterization of their photocatalytic properties. Thin Solid Films.

[23] Zhang J.K., Chen C.Q., Chen S., Hu Q.M., Gao Z., Li Y.Q., Qin Y. (2017). Highly dispersed Pt nanoparticles supported on carbon nanotubes produced by atomic layer deposition for hydrogen generation from hydrolysis of ammonia borane. Catal. Sci. Technol..

[24] Lin C.J., Yu Y.H., Liou Y.H. (2009). Free-standing TiO_2_ nanotube array films sensitized with CdS as highly active solar light-driven photocatalysts. Appl. Catal. B Environ..

[25] El-Hendawy A.N.A. (2003). Influence of HNO_3_ oxidation on the structure and adsorptive properties of corncob-based activated carbon. Carbon.

[26] Pradhan B.K., Sandle N.K. (1999). Effect of different oxidizing agent treatments on the surface properties of activated carbons. Carbon.

[27] Aarik J., Aidla A., Mandar H., Uustare T. (2001). Atomic layer deposition of titanium dioxide from TiCl_4_ and H_2_O: Investigation of growth mechanism. Appl. Surf. Sci..

[28] Puurunen R.L. (2005). Surface chemistry of atomic layer deposition: A case study for the trimethylaluminum/water process. J. Appl. Phys..

[29] Zhang Y.C., Guerra-Nunez C., Utke I., Michler J., Rossell M.D., Erni R. (2015). Morphology and crystallinity control of ultrathin TiO_2_ layers deposited on carbon nanotubes by temperature-step atomic layer deposition. J. Phys. Chem. C.

[30] Kočí K., Obalová L., Matějová L., Plachá D., Lacný Z., Jirkovský J., Šolcová O. (2009). Effect of TiO_2_ particle size on the photocatalytic reduction of CO_2_. Appl. Catal. B Environ..

[31] George S.M. (2010). Atomic layer deposition: An overview. Chem. Rev..

[32] Barka N., Qourzal S., Assabbane A., Nounah A., Ait-Ichou Y. (2008). Factors influencing the photocatalytic degradation of Rhodamine B by TiO_2_-coated non-woven paper. J. Photochem. Photobiol. A.

[33] Wang C.C., Hsueh Y.C., Su C.Y., Kei C.C., Perng T.P. (2015). Deposition of uniform Pt nanoparticles with controllable size on TiO_2_-based nanowires by atomic layer deposition and their photocatalytic properties. Nanotechnology.

[34] Rosseler O., Shankar M.V., Du M.K.-L., Schmidlin L., Keller N., Keller V. (2010). Solar light photocatalytic hydrogen production from water over Pt and Au/TiO_2_ (anatase/rutile) photocatalysts: Influence of noble metal and porogen promotion. J. Catal..

[35] Kiwi J., Gratzel M. (1984). Optimization of conditions for photochemical water cleavage-aqueous Pt/TiO_2_ (anatase) dispersions under ultraviolet-light. J. Phys. Chem..

[36] Sadeghi M., Liu W., Zhang T.G., Stavropoulos P., Levy B. (1996). Role of photoinduced charge carrier separation distance in heterogeneous photocatalysis: Oxidative degradation of CH_3_OH vapor in contact with Pt/TiO_2_ and cofumed TiO_2_-Fe_2_O_3_. J. Phys. Chem..

[37] Wang W.N., An W.J., Ramalingam B., Mukherjee S., Niedzwiedzki D.M., Gangopadhyay S., Biswas P. (2012). Size and structure matter: enhanced CO_2_ photoreduction efficiency by size-resolved ultrafine Pt nanoparticles on TiO_2_ single crystals. J. Am. Chem. Soc..

[38] Wang C.C., Kei C.C., Perng T.P. (2011). Fabrication of TiO_2_ nanotubes by atomic layer deposition and their photocatalytic and photoelectrochemical applications. Nanotechnology.

[39] Li X.Z., Li F.B. (2001). Study of Au/Au^3+^-TiO_2_ photocatalysts toward visible photooxidation for water and wastewater treatment. Environ. Sci. Technol..

[40] Li X.Z., Li F.B., Yang C.L., Ge W.K. (2001). Photocatalytic activity of WOx-TiO2 under visible light irradiation. J. Photochem. Photobiol. A.

[41] Yu J.C., Yu J.G., Ho W.K., Jiang Z.T., Zhang L.Z. (2002). Effects of F-doping on the photocatalytic activity and microstructures of nanocrystalline TiO_2_ powders. Chem. Mater..

[42] Isaifan R.J., Ntais S., Couillard M., Baranova E.A. (2015). Size-dependent activity of Pt/yttria-stabilized zirconia catalyst for ethylene and carbon monoxide oxidation in oxygen-free gas environment. J. Catal..

[43] Ciftci A., Ligthart D., Hensen E.J.M. (2015). Influence of Pt particle size and Re addition by catalytic reduction on aqueous phase reforming of glycerol for carbon-supported Pt(Re) catalysts. Appl. Catal. B Environ..

[44] Feng X.J., Sloppy J.D., LaTemp T.J., Paulose M., Komarneni S., Bao N.Z., Grimes C.A. (2011). Synthesis and deposition of ultrafine Pt nanoparticles within high aspect ratio TiO_2_ nanotube arrays: Application to the photocatalytic reduction of carbon dioxide. J. Mater. Chem..

[45] Yoo J., Altomare M., Mokhtar M., Alshehri A., Al-Thabaiti S.A., Mazare A., Schmuki P. (2016). Photocatalytic H_2_ generation using dewetted Pt-decorated TiO_2_ nanotubes: Optimized dewetting and oxide crystallization by a multiple annealing process. J. Phy. Chem. C.

[46] Kim G.J., Kwon D.W., Hong S.C. (2016). Effect of Pt particle size and valence state on the performance of Pt/TiO_2_ catalysts for CO oxidation at room temperature. J. Phy. Chem. C.

